# Quality-checking a novel “fact sheet” on ghostly episodes

**DOI:** 10.3389/fpsyg.2025.1585437

**Published:** 2025-07-01

**Authors:** Brandon Jon Massullo, James Houran, Alex Escolá Gascón, Ciarán O’Keeffe, Kenneth Graham Drinkwater, Neil Dagnall

**Affiliations:** ^1^Wooster Community Hospital, Wooster, OH, United States; ^2^Integrated Knowledge Systems, Dallas, TX, United States; ^3^Comillas Pontifical University, Madrid, Spain; ^4^Buckinghamshire New University, High Wycombe, United Kingdom; ^5^Manchester Metropolitan University, Manchester, United Kingdom

**Keywords:** encounter experiences, fact-checking, information sheet, public education, scientific literacy, sense-making

## Abstract

**Introduction:**

‘Apparitions, hauntings, and poltergeists’ are universally reported phenomena with significant psychological and social implications. Despite their prevalence, the scientific study of these anomalous experiences remains fragmented, and misinformation is widespread. To address this gap, a resource titled “Fact Sheet: Ghostly Episodes at a Glance” was developed to provide an evidence-based summary for educational and clinical use.

**Methods:**

This preliminary study evaluated the Fact Sheet’s content validity through an AI-based verification procedure. Additionally, we assessed its accessibility, utility, and global favorability among four groups: lay percipients, lay non-percipients (information-seekers), clinical practitioners, and amateur paranormal investigators (information-providers).

**Results:**

The Fact Sheet demonstrated strong content validity, accessibility, usefulness, and favorability across all groups. However, participants suggested refinements to enhance readability and contextual depth. Statistical analysis revealed small but significant differences in global favorability scores, with information-providers more strongly endorsing the resource than information-seekers.

**Conclusion:**

These findings underscore the importance of scientifically-grounded, accessible resources in educating diverse audiences about anomalous experiences. The study highlights the value of data-driven public education in this domain and offers specific recommendations for improving future iterations of the Fact Sheet to boost engagement and comprehension in both educational and clinical settings.

## Introduction

Encounters with ostensible spirits or non-human entities are central to many religio-spiritual traditions and practices (Plante and Schwartz, 2021; Santos and Michaels, 2022; Wilt et al., 2022). Their relevance also reaches secular contexts (Goldstein et al., 2007; Hill et al., 2018; Houran and Lange, 2001), with studies (e.g., Haraldsson, 1985; Laythe et al., 2018; Ross and Joshi, 1992) consistently indicating that a significant percentage of the general population has experienced ‘ghosts, hauntings, or poltergeists’ (collectively termed ‘ghostly episodes’ in this paper). For example, a large survey by the Pew Research Center (2009) found that 18% of Americans reported having seen or been in the presence of a ghost. Similarly, Moore’s (2005) survey indicated that around one-third of Americans believe in ghosts, with 37% reporting personal experiences that they interpreted as supernatural encounters. McClenon (2012) similarly found that 40% of respondents in a community survey had perceived an “apparition.” Another large-scale study by the Association for the Scientific Study of Anomalous Phenomena (ASSAP) found that 40% of UK respondents reported experiences they considered to be hauntings or encounters with ghosts (Castro et al., 2014). Poltergeist-like disturbances featuring physical anomalies—e.g., percussive knockings or objects displacements (Dullin, 2024)—are less common but still reported (Houran et al., 2019). Some averaged statistics (Ross and Joshi, 1992; YouGov, 2022) suggest that approximately 12% of survey respondents had encountered unusual physical events they interpreted as poltergeist activity. Overall, these findings suggest that belief in, and experiences of, ghostly episodes are relatively widespread across different cultural contexts, highlighting an area of common curiosity and personal significance for many people (Goldstein et al., 2007; Hill et al., 2018; Houran and Lange, 2001).

The deeply emotional or psychological effects that ghostly episodes often elicit (Coelho et al., 2021; Evrard et al., 2021; Houran et al., 2022) can motivate percipients to seek academic or clinical support with understanding the nature or meaning of their experiences. Unfortunately, many lay-oriented websites, podcasts, and books sensationalize the topic or provide information of either inconsistent or dubious quality (Hill, 2017; Hill et al., 2018; Potts, 2004). For instance, many sources use various vernaculars to claim incorrectly that ghostly episodes have been ‘scientifically-validated’ as being ‘paranormal or demonic’ phenomena. We think that these assertions are fundamentally unethical for promoting or confirming *emotion-based* beliefs versus representing *evidence-based* conclusions from peer-reviewed research (see, e.g., Andrade, 2017). Such proclamations also can heighten people’s distress by fueling their pre-existing fears or anxieties about the ontological reality of supernatural forces (cf. de Oliveira-Souza, 2018; Lange and Houran, 1999). These circumstances—in tandem with a modern case study of a help-seeking ‘haunted person’—encouraged Houran et al. (2024) to develop a Fact Sheet promoting awareness and responsible education on the topic of ghostly episodes. Accordingly, their tool aims to normalize versus pathologize these phenomena in line with the person-centered philosophy of modern clinical approaches to anomalous experiences (Hastings, 1983; Rabeyron, 2022; Roxburgh and Evenden, 2016a; Taves and Barlev, 2023; Woods and Wilkinson, 2017).

In particular, fact or information sheets are concise, easy-to-read resources that provide essential information on specific topics, thereby helping to promote awareness and education among diverse audiences. By summarizing key facts and presenting them in an organized way, information sheets simplify complex topics and enable users to better understand and remember pertinent data or associated recommendations (Peters et al., 2007). Their simple and direct format, often including bullet points, graphics, or charts, helps to convey quickly main ideas without overwhelming the reader with too many details (Houts et al., 2006). Fact Sheets also are a practical way to raise awareness of particular issues, because they can be shared widely across digital and print formats and thus effectively reach a broad audience. This ease of distribution allows individuals, organizations, and communities to stay informed on important issues, which can encourage positive actions related to health, environmental, or social topics (Katz et al., 2005). And since Fact Sheets are often created by trusted experts or institutions, they are generally viewed as a reliable and valuable resource for education and advocacy (Sun et al., 2019).

### The present research

Outdated or inaccurate information can lead to ineffective or harmful practices, which compromises client safety and trust in healthcare educators or providers (Bero et al., 1998). Quality-checking clinical and educational resources is essential to ensure that authorities rely on accurate, current information that supports effective decision-making and patient care. Clinical resources are foundational in guiding diagnosis, treatment plans, and patient interactions, so their accuracy can directly impact patient outcomes (Schulz and Grimes, 2005). Moreover, clinical resources that undergo thorough quality checks are more likely to reflect current research, evidence-based practices, and standardized guidelines. This supports consistent standards across different healthcare or educational settings (Shojania and Grimshaw, 2005).

Quality checks often involve verifying that information sources have been peer-reviewed or validating clinical recommendations against recent academic literature. This exercise not only enhances the credibility of clinical resources but also supports practitioners in maintaining professional competence (Carman et al., 2013). Therefore, quality assurance of clinical and educational information is critical to promote safety and excellence in educational or therapeutic delivery. Accordingly, we quality-checked Houran et al.’s (2024) “Fact Sheet: Ghostly Episodes at a Glance” (referred throughout this paper as simply “Fact Sheet”) in four respects: (a) validate its content against independent, peer-reviewed literature, (b) calculate its readability metrics to gauge its general accessibility, (c) assess the reactions of different target audiences to evidence-based information that specifically aims to demystify the topic, and (d) gain insights from different target audiences about potential improvements for future versions.

## Method

### Transparency and openness

Our study’s design, analysis, and research materials were not pre-registered, but the protocol was reviewed and approved by the Ethics Committee at Integrated Knowledge Systems. Moreover, we strived to follow the Journal Article Reporting Standards (Kazak, 2018) and thus describe how we determined our research samples, all data exclusions (if any), specific research questions, applicable manipulations, and all measures and data abstractions.

### Fact Sheet

Houran et al.’s (2024, pp. 200–202) “Fact Sheet: Ghostly Episodes at a Glance” (cf. Appendix A) is a 1,187-word resource developed by a multidisciplinary panel (Bertens et al., 2013) with expertise in quali-quantitative research across anomalistics and the social sciences. That team sourced key questions to answer in the Fact Sheet via informal conversations with research colleagues and known percipients of ghostly episodes. Then they used an iterative process of internal discussions and language refinements to produce the final version of the question-and-answer set that we evaluate here. Its content was not explicitly referenced or justified in its original source, although it drew heavily from recent research on the concept of Haunted People Syndrome (HP-S) (Laythe et al., 2021, 2022), combined with the results or conclusions from modern integrative works on ghostly episodes from parapsychological perspectives that were cited in the Fact Sheet. There can be various conventional explanations for one-off reports of ‘entity encounters’ or ‘haunted houses’ (Dagnall et al., 2020; Houran, 1997; Nickell, 2012), but HP-S specifically describes ghostly episodes recurrently manifesting to certain individuals as an interactionist phenomenon emerging from heightened somatic-sensory sensitivities that are stirred by ‘dis-ease’ states (i.e., when a person’s normal state of ‘ease’ becomes markedly disrupted or imbalanced), contextualized with paranormal belief or other sense-making mechanisms, and reinforced via perceptual contagion or threat-agency detection.

### Respondent groups

We surveyed individuals spanning four distinct convenience samples that represented target audiences for the Fact Sheet, with two comprising ‘information-seekers’ and another two being ‘information-providers.’ We recruited these diverse groups via multi-prong approaches as described below. Note that our minimum sample was only 24 respondents per group, which some authors contend is more than adequate for certain sentiment studies (e.g., Guest et al., 2006). This also parallels other researchers who used smaller, targeted groups to investigate various issues in clinical settings ranging from spirituality (e.g., Eksi et al., 2016) to drug administrations (e.g., Syroid et al., 2002):

1. *Lay percipients.* Data derived from 8 men and 16 women (*M*_age_ = 47.5, *SD* = 9.98, range = 28–68 yrs) from the USA (*n* = 4), UK (*n* = 18), Portugal (*n* = 1) and UAE (*n* = 1), who were recruited via an email and social media outreach campaign.
2. *Lay non-percipients.* Data derived from 10 men, 17 women, and 1 respondent who preferred not to disclose gender (*M*_age_ = 50.9 yrs., *SD* = 9.36, range = 30–75 yrs) from the USA (*n* = 6), UK (*n* = 13), Austria (*n* = 1), Denmark (*n* = 1), Australia (*n* = 1), Ireland (*n* = 1), Iceland (*n* = 1), Kenya (*n* = 1), Wales (*n* = 1) and Canada (n = 2), who were recruited via an email and social media outreach campaign.
3. *Clinical practitioners*. Data derived from 7 men and 23 women (*M*_age_ = 42.6 yrs., *SD* = 11.47, range = 27–72 yrs) who were recruited via email or personal communication. This US-based sample includes an advanced practice registered nurse (*n* = 1), psychiatrists (*n* = 2), therapists (mental health, trauma, and marriage-family; *n* = 5), social workers (hospital and hospice; *n* = 5), Licensed Independent Social Workers (LISW; *n* = 4), mental health counselors (*n* = 12), and a joint social-worker and mental health counselor (*n* = 1).
4. *Self-styled paranormal researchers (or ‘ghost-hunters’)*. Data derived from 20 men and 14 women (*M*_age_ = 49.5 yrs., *SD* = 7.86, range = 32–66 yrs) from the USA (*n* = 28), UK (*n* = 2), Australia (*n* = 2), Canada (*n* = 2) who were recruited via direct email or personal communication.

### Questionnaire

In addition to indicating their Age, Gender, and Country of Origin, the respondents completed five quality-related items administered in a standardized order and involving a mix of Likert rating scales and open-ended questions: (1) *Accessibility*: On a scale of 1 to 4, how easy was it to understand the information on the Fact Sheet? [1 = Very difficult, 2 = Somewhat difficult, 3 = Somewhat easy, 4 = Very easy]; (2) “Did you experience any difficulties accessing or reading the Fact Sheet (e.g., font size, layout, terminology)? Please explain; (3) *Utility*: How well did the Fact Sheet help you understand the topic it covers? [1 = Very unhelpful, 2 = Somewhat unhelpful, 3 = Somewhat helpful, 4 = Very helpful]; (4) What information, if any, do you feel is missing from the Fact Sheet that would improve its usefulness?; and (5) *Global Favorability*: How likely are you to recommend this Fact Sheet to someone looking for information on this topic? [1 = Very unlikely, 2 = Somewhat Unlikely, 3 = Somewhat Likely, 4 = Very likely]. This latter index follows from the popular Net Promoter Score (NPS) approach. NPS is a clear metric that many businesses use to assess consumer satisfaction and loyalty. It centers around a single, key question: “How likely are you to recommend our product or service to a friend or colleague?” Its simplicity and ability to provide actionable insights have made NPS a widely adopted measure in customer experience management (Reichheld, 2003). We drafted the three metrics above specifically for this study, so there are no prior psychometric data to report.

### Procedure

Our quality-check involved two complementary exercises. First, we worked as an expert panel (Bertens et al., 2013) to validate formally the Fact Sheet’s key statements against recent empirical literature. This included a rapid-type ‘critical review’ that considered our own work and independent studies alike. Unlike systematic reviews that involve exhaustive searches and long processing times, rapid reviews use targeted strategies for quickly identifying and synthesizing relevant literature to inform decision-making or research development (e.g., Tricco et al., 2017). The heading questions listed in the Fact Sheet were used as prompts in the AI language programs Consensus (Consensus AI, n.d.) and Co-Pilot (GitHub, n.d.). Further prompts included the key statements listed in Column 1 (effectively summary themes). We instructed both programs to provide academic references to support the answers. These were compared to the critical review references, which were confirmed in several cases. Any additional relevant references sourced by the AI programs were added to the list of empirical literature. Table 1 therefore presents a selection of this dually confirmed literature.

**Table 1:** AI-based content validation of the “Fact Sheet Ghostly Episodes.”

Key statement, finding, or conclusion	Authors’ supporting works	Independent supporting works
Are ghosts, hauntings, and poltergeists real? 1. Common phenomenon: Ghostly episodes, including ghosts, haunted houses, and poltergeist disturbances, share common principles and can deeply affect witnesses emotionally or psychologically. 2. Scientific debate: Scientists debate the nature of these anomalies, with some suggesting spirits, others attributing them to the psychic abilities of living people, and skeptics pointing to natural causes. 3. Lack of comprehensive explanation: While the general consensus is that these experiences are linked to the actions or psychology of living people, science currently lacks a completely proven solution for all aspects of ghostly episodes.	Hill et al. (2018), Dagnall et al. (2020), Houran and Lange (2001)	Alvarado and Zingrone (1995), Barrett (1911), Holzer (1965), Maher (2015), Maraldi (2017)
Who experiences these phenomena? 1. Hyper Sensitivities: Individuals with heightened awareness of their environment and bodily functions. 2. Blended Perceptions: Confusion between external information and internal sensations. 3. Multiple Sensitivities: Presence of chemical, emotional, psychological, or social sensitivities. 4. Mysterious Experiences: Reporting of various unexplained events beyond ghost or poltergeist disturbances.	Houran et al. (2023), Laythe et al. (2022), Laythe et al. (2021), Laythe et al. (2018), O’Keeffe et al. (2019), Ventola et al. (2019)	Becker (2020), Dagnall et al. (2010), Escolà-Gascón (2020), Langston et al. (2020), McAndrew (2020), Rabeyron and Loose (2015), Sangha (2020)
Are these phenomena dangerous? 1. Psychological Distress: Episodes are often unpredictable and unmanageable, causing mental stress. 2. Questioning Beliefs: The mysterious nature of episodes leads some to question their religious beliefs and sense of reality. 3. Physical Events: Rare occurrences of physical damage, such as objects being thrown or witnesses getting scratches. 4. Minimal Immediate Danger: Most episodes result in mental or spiritual anxiety rather than physical harm.	Houran et al. (2019, 2022), Ventola et al. (2019)	Dullin (2024), Lincoln and Lincoln (2015), de Oliveira-Souza (2018), Playfair (1980)
Can these phenomena be controlled or stopped? 1. Interventions: Efforts by paranormal investigators, religious leaders, or psychic mediums. 2. Varied Success Rates: Different outcomes from interventions, including cessation, temporary relief, intensification, or no effect. 3. Statistical Findings: Specific percentages of success, temporary relief, intensification, and no effect. 4. Psychological Support: The potential role of interventions in providing comfort and psychological support rather than addressing paranormal activity directly.	Laythe et al. (2022), Laythe et al. (2021)	Giordan and Possamai (2018), Palmer and Hastings (2013), Sanford (2016), Storm and Goretzki (2021)
What do skeptics say? 1. Skeptical Approach: Reasonable doubt and questioning of claims or beliefs. 2. Common Explanations: Fraud, psychological factors, and misinterpretations of natural events. 3. Acknowledgment of Complexity: Some cases are difficult to explain with current scientific knowledge. 4. Occam’s Razor: Preference for the simplest explanation with the fewest assumptions.	Dagnall et al. (2020), Hill et al. (2018, 2019), Jawer et al. (2020)	Bering et al. (2021), Castle (1991), Dean et al. (2022)
What should I do if my house seems haunted? 1. Varied Reactions: Some people find living with a “ghost” intriguing or fun, while others feel annoyed or threatened. 2. Seeking Knowledge: Those intrigued may want to learn more about the phenomena. 3. Professional Guidance: People feeling threatened are encouraged to consult trusted professionals like psychology professors or clergy. 4. Scientific Consultation: For intense cases, recommendations include consulting credible scientific organizations like the Society for Psychical Research and the Parapsychological Association. 5. Avoiding Amateurs: Advising against seeking help from unvetted ghost-hunting groups or amateur paranormal researchers.	Baker and O’Keeffe (2007), Laythe et al. (2022)	Clausman (1947), Ironside (2018), Rabeyron (2022)
Where can I find more reliable information? 1. Unreliable Sources: Popular media often provides unreliable information about ghostly episodes. 2. Anecdotal Evidence: Reliance on personal beliefs and sensationalism rather than empirical evidence. 3. Entertainment Over Accuracy: Prioritization of entertainment value leads to exaggeration and embellishment of stories or research findings.	Houran and Lange (2001), Laythe et al. (2022)	Parsons (2015, 2018)

Second, the target audiences rated the accessibility, utility, and global favorability of the Fact Sheet using a standardized survey. A personal outreach campaign that included snowball sampling, as appropriate, helped to ensure that respondents met the inclusion criteria for this research. To clarify, personal outreach campaigns use direct appeals to selected individuals, often through personalized emails or social media messages, to invite them to participate in research. This tailored approach tends to increase response rates, as the personalized nature of the outreach can make respondents feel more valued and engaged (Groves et al., 2009). And because participants in personal outreach campaigns are often selected based on specific criteria, the resulting data can better represent a targeted audience, which is particularly useful when aiming for precision in demographic or behavioral data (Dillman et al., 2014). In particular, we emailed the Fact Sheet and our questionnaire to respondents across the four groups.

## Results

### Content validation

Table 1 supports the Fact Sheet’s major statements or conclusions (Column 1) with two or more peer-reviewed works. The representative lists of supporting literature in Column 2 and 3 did not derive from selective reporting, however, as both the Consensus and Copilot AI programs similarly validated the accuracy of the key statements asserted in the sheet. The studies cited from the AI rapid-type critical literature review include the authors’ own recent works and independent sources. Moreover, we should emphasize that both AI programs provided a mix of skeptical and sympathetic literature on ghostly episodes.

### Statistical preliminaries

**Figure 1:**
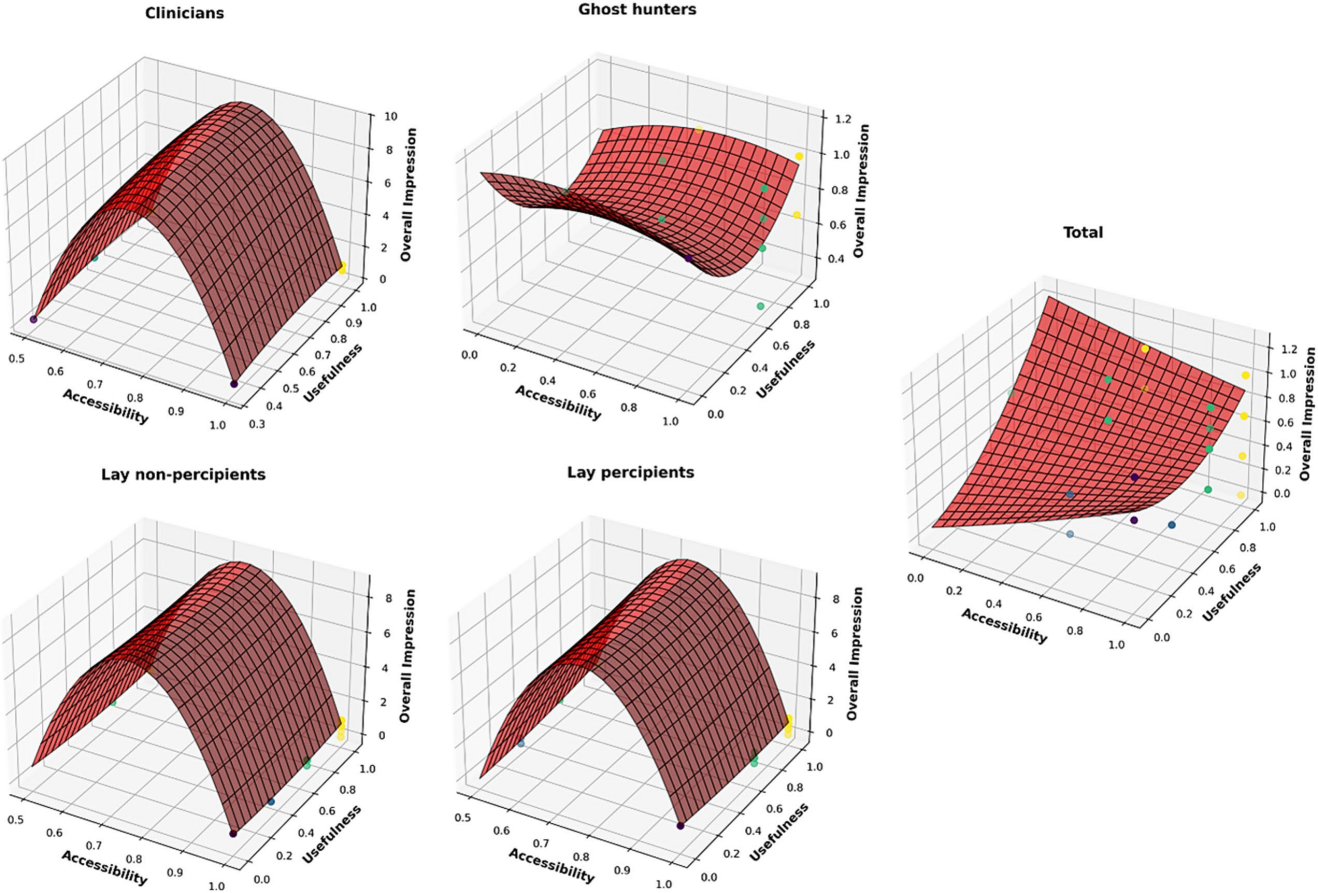
Curvilinear functions of the Accessibility, Usefulness, and Global Favorability metrics for the “Fact Sheet.” The relationship structures show quadratic interdependencies with upward trends.

We measured the Fact Sheet’s ‘Accessibility, Utility, and Global Favorability’ using a common Likert scale (maximum possible score of 4). Table 2 shows that all the mean scores in the present samples were close to this upper limit, indicating that the four audience groups perceived the content quality quite positively. Moreover, we conducted correlational analyses among the three metrics using curvilinear functions. Figure 1 illustrates the trends of these functions, with alpha curves adjusted to a visibility of 0.60 using the *Python* programming language (Python Software Foundation, 2023). The parameters obtained for reproducing these functions were as follows (in order): (a) Ghost-Hunters: −0.397, 1.25, 0.238, 0.262, −1.536, and 1.135; (b) Clinicians: −1.632, −8.155, −3.942, 7.447, 13.839, and −17.435; (c) Lay Percipients: −125.776, 0.631, −2.637, 190.641, 1.793, and −63.894; (d) Lay Non-Percipients: −124.714, 0.744, 0.470, 186.548, −0.975, and −61.266; and (e) Total: 0.064, 0.822, −0.983, 0.571, 0.307, and 0.097. Overall, the curvilinear structures were parabolic and upward-trending, accounting for up to 40% of the total variance.

**Table 2:** Descriptive statistics and content quality analysis.

Variables	Groups	*M*	*SD*	Fisher’s *F*	F *p*-values	χ^2^	χ^2^ *p*-values	BF_10_
Accessibility	Ghost hunters	3.82	0.459	0.252	0.860	1.10	0.776	0.0623 *P*(*H*_1_|*D*) = 5.9%
	Clinicians	3.77	0.430					
	Lay percipients	3.79	0.415					
	Lay non-percipients	3.86	0.356					
Usefulness	Ghost hunters	3.59	0.657	1.45	0.231	5.46	0.141	0.238 *P*(*H*_1_|*D*) = 19.3%
	Clinicians	3.80	0.551					
	Lay percipients	3.46	0.833					
	Lay non-percipients	3.46	0.793					
Global Favorability	Ghost hunters	3.56	0.746	4.28	0.007** (ω^2^ = 7.8%)	13.8	0.003** (ε^2^ = 12%)	5.60 *P*(*H*_1_|*D*) = 84.9%
	Clinicians	3.73	0.583					
	Lay percipients	3.13	0.992					
	Lay non-percipients	3.14	0.803					

SD, standard deviation; BF_10_, Bayes factor in favor of alternative hypothesis, using an equiprobable a priori distributions (50%) for null and alternative hypothesis; and P(H_1_|D) = Probability that the prior distribution assigned to the model (H1) adequately fits the observed data. The post hoc multiple comparison tests for the variable Overall Impression yielded significant results only for the mean difference of 3.56–3.13 (*p* = 0.029 < 0.05), with a standardized effect size of 0.756. The difference between 3.73 and 3.56 was also significant (*p* = 0.032 < 0.05), with a standardized effect size of 0.779.

The functions of the Clinicians, Lay Non-Percipients, and Lay Percipients overall exhibited consistent structural patterns, suggesting that these groups interpreted the content and applications of the Fact Sheet in a relatively homogeneous manner and with minimal conceptual discrepancies. The total 3D correlation in Figure 1 revealed an upward trend, indicating positive interrelations among the three metrics of content quality. This was further supported by Kendall’s *τ*-b linear correlations, which ranged from 0.20 to 0.40. The hypothesis tests in Table 1, the mean scores approaching the maximum rating of 4, and the three-dimensional graphical representations collectively provide robust evidence for the conceptual clarity and functional validity of the Fact Sheet.

### Accessibility metrics

The metrics in Table 3 indicate that the current version of the Fact Sheet is most suited to readers with a college-level or higher reading proficiency (United States standards), requiring some advanced vocabulary knowledge, strong comprehension skills, and experience with complex sentence structures. It may not be easily or uniformly accessible to the general public or readers with lower literacy levels, unless it is further explained by, or discussed in consultation with, educated researchers or practitioners. Still, both groups of information-seekers rated the accessibility of the content quite high, i.e., Lay Percipients (*M* = 3.79) and Lay Non-Percipients (*M* = 3.86). The perceived accessibility of the content also was on par between the information-seekers (aggregated *M* = 3.83) and information-providers (aggregated *M* = 3.80).

**Table 3:** Readability analysis of the “Fact Sheet Ghostly Episodes” via Scott’s (2024) software.

Metric	Definition	Score	Interpretation
Flesch Reading Ease (Flesch, 1948)	Scores on a 0–100 scale where higher scores mean easier readability. Scores above 60 are generally considered easily readable for most audiences.	30	With a low score, this text falls in the “difficult” range, suggesting it may be challenging to read and understand without advanced reading skills.
Flesch–Kincaid Grade Level (Kincaid et al., 1975)	Estimates the U.S. grade level necessary to understand the text. Lower scores (e.g., 6–8) indicate that the text is accessible to middle school readers, while higher scores suggest a more complex text.	13.44	This score indicates that the text is best suited for readers at a college freshman level or higher, implying a need for advanced literacy to fully comprehend the content.
Gunning Fog Index (Gunning, 1952)	Indicates the number of years of education needed to understand the text at first read.	16.3	This score suggests the text would be understandable to someone with at least 16 years of formal education, meaning a senior college level, reflecting high sentence complexity and vocabulary.
SMOG Index (McLaughlin, 1969)	Calculates reading level based on the number of complex words, ideal for assessing comprehension difficulty.	11.84	This index suggests the text is accessible to readers with at least a 12th-grade reading level, suitable for upper high school readers but still relatively complex.
Automated Readability Index (Smith and Senter, 1967)	Similar to other grade-level indices, estimating the minimum age required to understand the text.	14.52	This indicates a readability level aligned with 14–15 years of education, typically sophomore to junior college level, reinforcing the need for advanced comprehension skills.
Coleman-Liau Index (Coleman and Liau, 1975)	Focuses on the number of characters, words, and sentences, also providing a grade-level estimate.	15.43	This index suggests that a reader would need at least 15 years of education to understand the text, indicating a difficulty level appropriate for college students or advanced readers.

### Utility metrics

Table 3 also shows that our groups of information-providers (aggregated *M* = 3.70) and information-seekers (aggregated *M* = 3.46) both rated the Fact Sheet as highly useful, though the former gave consistently higher ratings than the former in this respect. The open-ended feedback discussed outlines some probable reasons for this outcome, which involve issues with presenting technical information to a lay audience. Indeed, we observed no differences in the tool’s perceived utility across the Lay Percipients and Lay Non-Percipients.

### Global favorability metrics

Recall that the overall impression of the Fact Sheet was assessed via a one-item index of ‘global favorability’ (i.e., “How likely are you to recommend this ‘Fact Sheet on Ghostly Episodes’ to someone looking for information on this topic?” on a 1–4 scale). The information-providers (aggregated *M* = 3.65) and information-seekers (aggregated *M* = 3.14) both had good impressions of the tool, indicating they were “Somewhat Likely” to “Highly Likely” to recommend it as a resource to others. However, as reported below, we observed some small but statistically significant differences among the groups’ ratings.

### Group comparisons

Table 2 gives descriptive statistics and summarizes the analysis of differences on the metrics of Accessibility, Utility, and Global Favorability across the information providers (i.e., Clinicians vs. Ghost-Hunters) and information-seekers (i.e., Lay Percipients vs. Lay Non-Percipients). Ideally, no statistically significant differences emerge, as these could introduce variability and potential bias that may require further consideration. Although Accessibility and Usefulness showed no significant effects, we found some discrepancies on Global Favorability between Ghost-Hunters and both Clinicians and Lay Percipients. Multiple comparisons revealed that Clinicians scored significantly higher, which was not unexpected, given that the Fact Sheet was designed to align with professional needs in mental health and psychology.

To ensure a robust estimation, the Bayes Factor (BF) was included, with prior probabilities for the null and alternative hypotheses set at 50%. This approach integrates Bayesian hypothesis testing within the classical frequentist framework while maintaining the advantages of Bayesian inference. The BF values did not exceed 10, i.e., the commonly used threshold in this type of analysis due to its odds-based interpretation (Escolà-Gascón, 2022). This suggests that the observed significance for this metric should be considered marginal, as the statistical validity of the differences cannot be confidently established. This interpretation is further supported by effect size estimates based on explained variance (ω^2^ and ε^2^ coefficients), with a maximum effect size of 12%. The absence of significant differences is not necessarily problematic, as it may indicate that the Fact Sheet exhibits low inter-population variability. This, in turn, suggests that the tool’s structure is less susceptible to sociocultural biases.

### Suggested refinements

Visual inspection of the participants’ open-ended feedback, supplemented by a thematic analysis via the popular AI language program ChatGPT-4 (OpenAI, 2023), suggested four categories of recommended improvements to future versions of the Fact Sheet involving (a) Accessibility and Readability, (b) Content Completeness and Utility, (c) Audience Tailoring and Tone, and (d) Additional Topics of Interest. Table 4 summarizes this set of feedback from the four target groups. All the audiences consistently lauded the Fact Sheet’s professional and balanced approach to contextualizing ghostly episodes, but they likewise called for improved readability, emotional support, and practical advice tailored to the specific needs of each audience.

**Table 4:** Summary of open-ended feedback on refining the “Fact Sheet on Ghostly Episodes.”

Audience	Major themes
Paranormal investigators	Accessibility Clear Language: Use simple, straightforward language for easy readability, especially for non-specialist audiences. Improved Formatting: Break up complex or technical sections into bullet points. Use shorter paragraphs for easier scanning. Grammatical Corrections: Address minor grammar issues and adjust the flow for smoother readability. Simplify Technical Terms: Where possible, rephrase complex technical phrases to ensure accessibility. Cross-Platform Compatibility: Ensure the document’s formatting is compatible across various platforms (e.g., mobile, desktop). Multiple Formats: Offer the document in various formats like PDF or web versions to cater to different needs. Utility Simplified Language: Adapt complex sections to a lower reading level without losing meaning, using a reassuring tone, especially for emotionally distressed audiences. Enhanced Presentation: Incorporate bullet points, visuals, and concise paragraphs to boost engagement. Fill Information Gaps: Include definitions, examples, and practical steps. Add perspectives on medical, cultural, and alternative scientific views. Balanced Tone: Maintain scientific rigor while being sensitive to the personal and emotional aspects of paranormal experiences. Targeted Audience: Define the audience clearly (e.g., general readers, researchers, individuals experiencing phenomena) and tailor the content accordingly. General Suggestions The feedback highlights the Fact Sheet’s clarity and balanced tone. Further attention could be given to targeting specific audiences, offering practical advice, and providing emotional support for individuals facing paranormal experiences
Clinical practitioners	Accessibility Clear Content: Most users found the content easy to understand and accessible. Formatting: A suggestion was made to use bullet points for better readability. Utility Psychoeducation: There were requests for more information on differentiating hallucinations from paranormal experiences. Therapist Guidance: Interest in strategies for therapists managing paranormal concerns. Cultural & Historical Perspectives: A desire for inclusion of cultural and historical views on paranormal beliefs. General Suggestions Enhanced Readability: Incorporate bullet points to improve clarity. Information Evaluation: Add a section on how to assess the reliability of external information. Validating Language: Use language that validates diverse experiences to foster inclusivity. Audience Clarity: Clarify if the content is for clinicians or the general public. Content Adjustments: Prioritize free resources. Reorder sections for better flow and understanding.
Lay percipients	Accessibility User Experience: Most users did not report issues accessing or reading the content. Formatting Suggestions: Use bullet points, subheadings, and italics to improve readability. Enhance the presentation with color, graphics, and improved overall formatting. Utility Content Completeness: Most respondents felt that no critical information was missing. Suggestions for Clarity and Engagement: Include examples to clarify concepts and engage readers. Add historical context for ghostly experiences. Clarify the types of phenomena discussed (e.g., visual, acoustic, temperature changes). Explore multiple explanations for ghostly phenomena. Tone and Audience: Some felt the content was too academic and not tailored for a general audience. One comment noted that the content was not “interesting.” Additional Topics: Request for more discussion on the physiological effects of ghostly encounters.
Lay non-percipients	Accessibility User Experience: Most users had no issues accessing or reading the content. Several praised the content for being well-organized, informative, and well-written. Suggestions for Improvement: Shorten sections for brevity. Rename the Fact Sheet to “Summary of Current Research on Ghostly Episodes” to better reflect its overview nature, rather than focusing on “hard facts.” One commenter found the term “afflicted” offensive. Utility Content Completeness: Most respondents did not feel any critical information was missing. The Fact Sheet was considered a strong foundational overview. Requests for Additional Information: Clarifications on specific points, additional references, and further medical explanations for ghostly phenomena. A desire for more skeptical perspectives. Suggestions for Improvement: Discuss the negative impact and psychological distress of dismissing individuals’ ghostly experiences. Provide clearer distinctions between hauntings, poltergeists, and related phenomena. Specific Inquiry: One comment inquired about how to respond in the moment when encountering a ghost.

In particular, the amateur paranormal investigators recommended further simplifying the language, improving formatting with bullet points and shorter paragraphs, and offering multiple formats for accessibility. There was also suggestions to balance scientific rigor with emotional sensitivity. That is, some respondents thought that incorporating a gentler, more supportive or reassuring tone may enhance its impact or effectiveness, especially for percipients who are distressed about their anomalous experiences. Clinical practitioners expressed interest in more guidance on distinguishing paranormal experiences from hallucinations, strategies for therapists, and the inclusion of cultural and historical perspectives. Lay percipients sought content that is more engaging and less academic, with requests for examples, historical context, and clarification of different types of paranormal phenomena. They also wanted to explore physiological effects and multiple explanations for ghostly experiences. Lay non-percipients further suggested shortening sections for brevity, renaming the Fact Sheet for accuracy, and including more skeptical perspectives and clearer distinctions between various anomalous phenomena. Future efforts might thus strive to further simplify the current content or perhaps augment the text with images, tables, or figures to make the material more visually engaging (Nielsen and Loranger, 2006) or compatible with diverse learning styles (Clark and Paivio, 1991). Research indeed shows that people are more likely to remember information when it is paired with relevant images (McCrudden and Schraw, 2007). The Fact Sheet fits a single page when printed double-sided, though there might be room for some appropriate graphic(s) if the font size and content placement are adjusted.

Although not included as part of the participants’ suggested improvements, Appendix B provides our initial ‘children’s form’ of the Fact Sheet to address ghostly episodes or related fears reported by this vulnerable population. This version is certainly justified and should be helpful, because it is not uncommon for children of various ages to encounter ‘ghosts’ or other types of anomalous entities, including ‘deep’ imaginary friends that seemingly ‘come to life’ and exhibit a personality or will of their own (e.g., Drinkwater et al., 2024; Lange et al., 2023; Laythe et al., 2021; Little et al., 2021). Similarly, poltergeist-like disturbances often seem to focus on the presence of particular children or adolescents (for important discussions on this point, see Houran et al., 2022; Roll, 1977; Ventola et al., 2019). Muris et al. (2001) further reported that the vast majority of children they interviewed about nighttime anxieties referenced a fear of ghosts and monsters, which they attributed to negative information versus conditioning or modeling. Therefore, making the Fact Sheet accessible to young people who are naturally curious can help them to understand this topic (and their experiences, as applicable) in a way that is constructive and age appropriate. Child-friendly material also encourages early education, fosters critical thinking, and ensures that kids are not confused or misinformed by complex or misleading sources (Dwyer, 2023; Gilmour, 2024; Ku et al., 2023).

## Discussion

Information sheets and clear-cut summaries of parapsychological topics have certainly been published before now (e.g., Palmer et al., 1989; Van Dyke and Juncosa, 1973; Zingrone et al., 2015). Instructive, freely available examples include (a) *Psychology Today*’s online overview of parapsychology.,^1^ (b) the Society for Psychical Research’s *Psi Encyclopedia*^2^ with its accessible articles across a vast array of psi-related subjects, and (c) the Windbridge Research Center’s various Fact Sheets on ‘mediumship’ phenomena and ‘end-of-life’ experiences penned from a pro-paranormal perspective^3^. But ours is perhaps the first Fact Sheet for both professional and lay audiences that collates key scientific information about the often-sensationalized topic of ghostly episodes. Its content draws on current, independent studies in peer-reviewed journals, and the descriptions avoid ideological bias (pro or con) concerning the ontological reality of controversial mechanisms like putative psi (e.g., Cardeña, 2018) or postmortem survival of consciousness (e.g., Wahbeh et al., 2023). Accordingly, our Fact Sheet speaks fairly both to information-seekers who have had anomalous experiences or not, and to those who believe in the paranormal or not. Note the title of the Fact Sheet is intentionally simple and accessible given that research suggests shorter titles are easier to understand and increase reader engagement (Letchford et al., 2015; Paiva et al., 2012; Subotic and Mukherjee, 2014).

Some readers might question the need for this resource in routine educational or clinical practice, so two points are worth noting here. First, many practitioners are likely to interact with percipients of the ‘mystical or paranormal’ at some point. In particular, thin-boundary (or encounter-prone) individuals consistently report various clinically-relevant issues like mood swings, substance use, memory aberrations, nightmares, and night terrors (Houran et al., 2002; Houran and Thalbourne, 2003; Lange et al., 2000; Thalbourne et al., 2003a; Thalbourne et al., 2003b; Thalbourne et al., 2001; Thalbourne and Houran, 2005), as well as an array of non-ordinary cognitions or experiences typically attributed to the supernatural (Evans et al., 2019; Kumar and Pekala, 2001; McClenon, 2012; Rosen et al., 2023; Roxburgh et al., 2024; Simmonds-Moore, 2024; Swami et al., 2024). Second, practitioners may neither know about nor understand the scientific literature on ghostly episodes. This can effectively limit their ability to facilitate responsible education or sense-making with percipients, a consideration that likewise applies to self-styled paranormal investigators who often lack professional training or credentials in scientific research (Hill, 2017; Hill et al., 2019; Potts, 2004). Some authors have nonetheless proposed systems for people to assess the quality of information that they source on ghostly episodes (e.g., Laythe et al., 2022, pp. 229–231), but this is not equivalent to having a ready-made, integrative, and accessible summary of key findings in this domain.

Though not representing strong or consistently significant effects, our sample of information-*seekers* nonetheless tended to rate the utility and global favorability of the Fact Sheet slightly lower than the information-*providers*. This raises questions of potential ideological biases and associated mediators or moderators of the acceptance of (or resistance to) scientific findings on ghostly episodes—especially when information-seekers want validation that their experiences were truly paranormal (cf. Rabeyron, 2022). This tool therefore cautions clinicians who might expect that these experiences are wholly explained by current scientific models, as well as amateur ghost-hunters who might assume that these experiences are mostly parapsychological in nature. These issues further speak to the literature on misinformation, disinformation, and malinformation—terms are often used interchangeably, yet describe distinct types of false or harmful information based on their *intent* and *accuracy*. Misinformation stems from a lack of awareness, disinformation thrives on deceit, and malinformation exploits truth for ulterior motives (Lewandowsky et al., 2017; Wardle and Derakhshan, 2017). Kandel (2020) even proposed three grades of ‘information disorder’ with increasing severity. We draw on this system to speculate that most public misinformation about ghostly episodes is likely “Grade 1” (i.e., a milder form in which the individual shares false information without the intent of harming others), although some examples probably involve Grade 2, i.e., “… a moderate form in which the individual develops and shares false information with the intent of making money and political gain, but not with the intent of harming people (Kandel, 2020, p. 280).

Despite the Fact Sheet’s beneficial content and features, our results suggest that its utility is restricted as a ‘standalone’ resource for some audiences (cf. Clarke et al., 2024). This situation means that information-providers might better use the tool as a discussion sheet whereby information-seekers are walked through the content to ensure a full and fair understanding of the material. On the other hand, information-seekers with good levels of education or verbal comprehension should be able to consult the Fact Sheet ‘as is.’ Another key audience for the Fact Sheet apart from clinical practitioners and self-styled paranormal investigators could be ‘paranormal tours’ operators, who typically mesh history and folklore for commercial entertainment (Houran et al., 2020). We should mention here that attendees are more likely to recommend or return for future tours when they feel they are learning something of value (Hill, 2017). Indeed, many paranormal tourists are seeking an opportunity for personal growth or cultural exploration (Hanks, 2018). Incorporating credible information also addresses the ethical responsibilities of operators, as misleading tourists can introduce legal complications if they feel deceived (Sharpley, 2018). Accordingly, a balanced approach—combining authenticity with open-minded speculation—tends to captivate paranormal tourists far more effectively than simply hearing sensationalized ghost stories (cf. Tarlow, 2005).

We acknowledge other important limitations with this research, such as our use of single-item measures that are sometimes criticized on psychometric grounds (Allen et al., 2022). Moreover, the results derived from smaller samples with a restricted measurement of respondents’ demographic variables that could have influenced the quality ratings. The present findings should therefore be considered preliminary and in need of cross-cultural verification. It might also be useful to correlate impressions of the Fact Sheet with respondents’ education levels and duration in their respective roles as clinical practitioners or self-styled paranormal investigators, as applicable. Regarding potential moderators of the percipients’ ratings, it also could have been instructive to understand the intensity of their ghostly episodes as measured by Houran et al.’s (2019) Survey of Strange Events, or to measure the time elapsed since the percipients’ ghostly episodes occurred, which might lead to either embellished recollections of anomalous experiences (e.g., Lange et al., 2004) or interpretations that are skewed *for* or *against* the paranormal (e.g., Drinkwater et al., 2019). Finally, we gauged only the perceived quality of the content versus its educational or clinical impacts on its intended audiences (see, e.g., Lam et al., 2025). Future research should therefore include outcome studies to confirm the tool’s capacity to facilitate efficacious sense-making for percipients or those seriously interested in credible scientific information on this topic.

*Knowledge is power* as the saying goes (cf. Bacon, 1597-1996; Hobbes, 1668-1994). But when presented via sympathetic information sheets, knowledge also can serve as ‘permission slips’ for percipients to freely share their stories with practitioners or researchers. This is important since belief in paranormal and spiritual phenomena (including ghosts and non-human discarnate agents) often arises from lived experiences (Clarke, 1995; Cseh et al., 2024; Jackson et al., 2023), although percipients may be reluctant to discuss their experiences or beliefs for fear of being ridiculed or pathologized (Blinston, 2013; Mohr and Huguelet, 2004; Roxburgh and Evenden, 2016a, 2016b). Other times, percipients seek expert support for their fears of being hurt, going crazy, hurting someone else (i.e., a sense of responsibility toward others), or losing control (Siegel, 1986). We often find therefore that percipients are eager to share their accounts with interested professionals who are able to impart to them a sense of understanding, normalization, or contribution to science. It seems that both clinical and research approaches should correspondingly work in tandem to advance a holistic understanding of the nature or meaning of these often dramatic and even transformative occurrences. The reality is that ghostly episodes will likely never go away (Hill et al., 2018). Therefore, clinical and research professionals alike should become sufficiently educated to engage these reports with empathy and intellectual humility so that percipients may better understand and cope with this universal aspect of human experience.

## Data Availability

The raw data supporting the conclusions of this article will be made available by the authors, without undue reservation.

## Footnotes

^1^ https://www.psychologytoday.com/us/basics/parapsychology

^2^ https://psi-encyclopedia.spr.ac.uk/

^3^ https://www.windbridge.org/fact-sheets/

## References

[ref1] Allen M. S. Iliescu D. Greiff S. (2022). Single item measures in psychological science: a call to action [editorial]. Eur. J. Psychol. Assess. 38, 1–5. doi: 10.1027/1015-5759/a000699

[ref9001] Altay S. Berriche M. Acerbi A. (2023). Misinformation on misinformation: Conceptual and methodological challenges. Social Media Society, 9. doi: 10.1177/20563051221150412

[ref2] Alvarado C. S. Zingrone N. L. (1995). Characteristics of hauntings with and without apparitions: an analysis of published cases. J. Soc. Psych. Res. 60, 385–397.

[ref3] Andrade G. (2017). Is past life regression therapy ethical? J. Med. Ethics His. Med. 10:11. PMC5797677 29416831

[ref4] Bacon F. (1597-1996). Meditationes sacrae and human philosophy. Whitefish, Montana: Kessinger Publishing.

[ref5] Baker I. O’Keeffe C. (2007). Ethical guidelines for the investigation of haunting experiences. J. Soc. Psych. Res. 71, 216–229.

[ref6] Barrett W. F. (1911). “Hauntings and poltergeists” in Psychical research. ed. Barrett W. F. (London, England: Henry Holt and Co), 187–210.

[ref7] Becker M. E. (2020). Hauntings, history, and fieldwork: A sensitive’s journey. Bedford, Pennsylvania: Ghost Excavation Books, Inc.

[ref8] Bering J. Smith S. Stojanov A. Halberstadt J. Hughes R. (2021). The “ghost” in the lab: believers’ and non-believers’ implicit responses to an alleged apparition. Int. J. Psychol. Relig. 32, 214–231. doi: 10.1080/10508619.2021.1975400

[ref9] Bero L. A. Grilli R. Grimshaw J. M. Harvey E. Oxman A. D. Thomson M. A. (1998). Closing the gap between research and practice: an overview of systematic reviews of interventions to promote the implementation of research findings. BMJ 317, 465–468. doi: 10.1136/bmj.317.7156.465, PMID: 9703533 PMC1113716

[ref10] Bertens L. C. Broekhuizen B. D. Naaktgeboren C. A. Rutten F. H. Hoes A. W. van Mourik Y.. (2013). Use of expert panels to define the reference standard in diagnostic research: a systematic review of published methods and reporting. PLoS Med. 10:e1001531. doi: 10.1371/journal.pmed.1001531, PMID: 24143138 PMC3797139

[ref11] Blinston I. (2013). Disclosure of childhood spiritual encounter phenomena. J. Transp. Res. 5, 58–64.

[ref12] Cardeña E. (2018). The experimental evidence for parapsychological phenomena: a review. Am. Psychol. 73, 663–677. doi: 10.1037/amp0000236, PMID: 29792448

[ref13] Carman K. L. Dardess P. Maurer M. Sofaer S. Adams K. Bechtel C.. (2013). Patient and family engagement: a framework for understanding the elements and developing interventions and policies. Health Aff. 32, 223–231. doi: 10.1377/hlthaff.2012.1133 23381514

[ref14] Castle T. (1991). Contagious folly: “an adventure” and its skeptics. Crit. Inq. 17, 741–772. doi: 10.1086/448611

[ref15] Castro M. Burrows R. Wooffitt R. (2014). The paranormal is (still) normal: the Sociological implications of a survey of paranormal experiences in Great Britain. Sociol. Res. Online 19, 30–44. doi: 10.5153/sro.3355

[ref18] Clarke B. Alley L. J. Ghai S. Flake J. K. Rohrer J. M. Simmons J. P.. (2024). Looking our limitations in the eye: a call for more thorough and honest reporting of study limitations. Soc. Personal. Psychol. Compass 18. doi: 10.1111/spc3.12979, PMID: 40384257

[ref17] Clarke D. (1995). Experience and other reasons given for belief and disbelief in paranormal and religious phenomena. J. Soc. Psych. Res. 60, 371–384.

[ref16] Clark J. M. Paivio A. (1991). Dual coding theory and education. Educ. Psychol. Rev. 3, 149–210. doi: 10.1007/BF01320076

[ref19] Clausman R. (1947). What to do in a haunted house. Manuscripts 15:16.

[ref20] Coelho C. M. Zsido A. N. Suttiwan P. Clasen M. (2021). Super-natural fears. Neurosci. Biobehav. Rev. 128, 406–414. doi: 10.1016/j.neubiorev.2021.06.036, PMID: 34186152

[ref21] Coleman M. Liau T. L. (1975). A computer readability formula designed for machine scoring. J. Appl. Psychol. 60, 283–284. doi: 10.1037/h0076540

[ref22] Consensus AI. (n.d.). Consensus [AI-powered academic search engine]. Available online at: https://consensus.app (Accessed January 03, 2025).

[ref23] Cseh O. Karsai I. Szabo A. (2024). The relationship of life-changing spiritual experiences to current religious/spiritual attitudes and practices: a pilot study. Pastor. Psychol. 73, 227–238. doi: 10.1007/s11089-023-01120-9

[ref24] Dagnall N. Drinkwater K. Munley G. Parker A. Drinkwater K. (2010). Paranormal belief, schizotypy, and transliminality. J. Parapsychol. 74, 117–141.

[ref25] Dagnall N. Drinkwater K. O’Keeffe C. Ventola A. Laythe B. Jawer M. A.. (2020). Things that go bump in the literature: an environmental appraisal of “haunted houses”. Front. Psychol. 11:1328. doi: 10.3389/fpsyg.2020.01328, PMID: 32595577 PMC7304295

[ref27] Dean C. E. Akhtar S. Gale T. M. Irvine K. Grohmann D. Laws K. R. (2022). Paranormal beliefs and cognitive function: a systematic review and assessment of study quality across four decades of research. PLoS One 17:e0267360. doi: 10.1371/journal.pone.0267360, PMID: 35507572 PMC9067702

[ref26] de Oliveira-Souza R. (2018). Phobia of the supernatural: a distinct but poorly recognized specific phobia with an adverse impact on daily living. Front. Psych. 9. doi: 10.3389/fpsyt.2018.00590, PMID: 30505286 PMC6250805

[ref28] Dillman D. A. Smyth J. D. Christian L. M. (2014). Internet, phone, mail, and mixed-mode surveys: The tailored design method. 4th Edn. Hoboken, New Jersey: John Wiley & Sons Inc.

[ref29] Drinkwater K. Dagnall N. Houran J. Denovan A. O’Keeffe C. (2024). Structural relationships among mental boundaries, childhood imaginary companions, and anomalous experiences. Psychol. Rep. 127, 2717–2735. doi: 10.1177/00332941221123235, PMID: 35996314 PMC11529105

[ref30] Drinkwater K. Laythe B. Houran J. Dagnall N. O’Keeffe C. Hill S. A. (2019). Exploring gaslighting effects via the VAPUS model for ghost narratives. Austr. J. Parapsychol. 19, 143–179.

[ref31] Dullin E. (2024). A detailed phenomenology of poltergeist events. J. Sci. Expl. 38, 427–460. doi: 10.31275/20243263

[ref32] Dwyer C. P. (2023). An evaluative review of barriers to critical thinking in educational and real-world settings. J. Intelligence 11. doi: 10.3390/jintelligence11060105, PMID: 37367507 PMC10300824

[ref33] Eksi H. Takmaz Z. Kardas S. (2016). Spirituality in psychotherapy settings: a phenomenological inquiry into the experiences of Turkish health professionals. Spiritual Psychol. Counsel. 1, 89–108. doi: 10.12738/spc.2016.1.0005

[ref34] Escolà-Gascón Á. (2020). Researching unexplained phenomena: empirical-statistical validity and reliability of the multivariable multiaxial suggestibility Inventory-2 (MMSI-2). Heliyon 6:e04291. doi: 10.1016/j.heliyon.2020.e04291, PMID: 32671247 PMC7347654

[ref35] Escolà-Gascón Á. (2022). Handbook of statistics: Step-by-step mathematical solutions. New York, New York: McGraw-Hill Education.

[ref38] Evans J. Lange R. Houran J. Lynn S. J. (2019). Further psychometric exploration of the transliminality construct. Psychol. Conscious. Theory Res. Pract. 6, 417–438. doi: 10.1037/cns0000163

[ref39] Evrard R. Dollander M. Elsaesser E. Cooper C. E. Lorimer D. Roe C. (2021). Exceptional necrophanic experiences and paradoxical mourning: studies of the phenomenology and repercussions of frightening experiences of contact with the deceased. Psychiatr. Evol. 86, 799–824. doi: 10.1016/j.evopsy.2021.05.002

[ref40] Flesch R. (1948). A new readability yardstick. J. Appl. Psychol. 32, 221–233. doi: 10.1037/h0057532 18867058

[ref41] Gilmour T. (2024). Critical thinking and media literacy in an age of misinformation (v2). Am. Polit. Sci. Assoc. Millington, Tennessee. 13. doi: 10.33774/apsa-2024-bsmtn-v2

[ref42] Giordan G. Possamai A. (2018). The sociology of exorcism in late modernity. Cham, Switzerland: Palgrave Macmillan.

[ref43] GitHub. (n.d.). GitHub copilot [AI-based code completion tool]. Available online at: https://github.com/features/copilot (Accessed January 03, 2025).

[ref44] Goldstein D. E. Grider S. A. Thomas J. B. (2007). Haunting experiences: Ghosts in contemporary folklore. Logan, Utah: Utah State University Press.

[ref45] Groves R. M. Fowler F. J. Couper M. P. Lepkowski J. M. Singer E. Tourangeau R. (2009). Survey methodology. 2nd Edn. Hoboken, New Jersey: John Wiley & Sons Inc.

[ref46] Guest G. Bunce A. Johnson L. (2006). How many interviews are enough?: an experiment with data saturation and variability. Field Methods 18, 59–82. doi: 10.1177/1525822X05279903

[ref47] Gunning R. (1952). The technique of clear writing. New York: McGraw-Hill.

[ref48] Hanks M. (2018). Haunted heritage: The cultural politics of ghost tourism, populism, and the past. Walnut Creek, California: Left Coast Press. Routledge.

[ref49] Haraldsson E. (1985). Representative national surveys of psychic phenomena: Iceland, Great Britain, Sweden, USA, and Gallup’s multinational survey. J. Soc. Psych. Res. 53, 145–158.

[ref50] Hastings A. (1983). A counseling approach to parapsychological experience. J. Transpers. Psychol. 15, 143–167.

[ref51] Hill S. A. (2017). Scientifical Americans: The culture of amateur paranormal researchers. Jefferson, North Carolina: McFarland & Co.

[ref52] Hill S. A. Laythe B. Dagnall N. Drinkwater K. O’Keeffe C. Ventola A.. (2019). “Meme-spirited”: II. Illustrations of the VAPUS model for ghost narratives. Austr. J. Parapsychol. 19, 5–43.

[ref53] Hill S. A. O’Keeffe C. Laythe B. Dagnall N. Drinkwater K. Ventola A.. (2018). “Meme-spirited”: I. A VAPUS model for understanding the prevalence and potency of ghost narratives. Austr. J. Parapsychol. 18, 117–152.

[ref54] Hobbes T. (1668-1994). *Leviathan*: *With selected variants from the Latin edition of 1668*. Indianapolis, Indiana: Hackett Publishing Co, Inc.

[ref55] Holzer H. (1965). Ghosts I've met. Indianapolis, Indiana: Bobbs-Merrill.

[ref56] Houran J. (1997). Ambiguous origins and indications of “poltergeists”. Percept. Mot. Skills 84, 339–344. doi: 10.2466/pms.1997.84.1.339

[ref57] Houran J. Hill S. A. Haynes E. D. Bielski U. A. (2020). Paranormal tourism – market study of a novel and interactive approach to space activation and monetization. Cornell Hosp. Q. 61, 287–311. doi: 10.1177/1938965520909094

[ref58] Houran J. Kumar V. K. Thalbourne M. A. Lavertue N. E. (2002). Haunted by somatic tendencies: Spirit infestation as psychogenic illness. Mental Health Relig. Cult. 5, 119–133. doi: 10.1080/13674670210141061

[ref59] Houran J. Lange R. (2001). Hauntings and poltergeists: Multidisciplinary perspectives. Jefferson, North Carolina: McFarland & Co.

[ref60] Houran J. Lange R. Laythe B. Dagnall N. Drinkwater K. O’Keeffe C. (2019). Quantifying the phenomenology of ghostly episodes – part II: a Rasch model of spontaneous accounts. J. Parapsychol. 83, 25–46. doi: 10.30891/jopar.2019.01.03

[ref61] Houran J. Laythe B. Lange R. Hanks M. Ironside R. (2023). Immersive study of gestalt variables in uncanny geographies. J. Soc. Psych. Res. 87, 65–100.

[ref62] Houran J. Little C. Laythe B. Ritson D. W. (2022). Uncharted features and dynamics of the South Shields poltergeist. J. Soc. Psych. Res. 86, 129–164.

[ref63] Houran J. Massullo B. Drinkwater K. Dagnall N. (2024). Team analysis of a help-seeking “haunted person”. Austr. J. Parapsychol. 24, 155–202.

[ref64] Houran J. Thalbourne M. A. (2003). Transliminality correlates positively with self-reported aberrations in memory. Psychol. Rep. 96, 1300–1304. doi: 10.2466/pms.2003.96.3c.1300, PMID: 12929785

[ref65] Houts P. S. Doak C. C. Doak L. G. Loscalzo M. J. (2006). The role of pictures in improving health communication: a review of research on attention, comprehension, recall, and adherence. Patient Educ. Couns. 61, 173–190. doi: 10.1016/j.pec.2005.05.004 16122896

[ref66] Ironside R. (2018). Feeling spirits: sharing subjective paranormal experience through embodied talk and action. Text Talk 38, 705–728. doi: 10.1515/text-2018-0020

[ref67] Jackson J. C. Dillion D. Bastian B. Watts J. Buckner W. DiMaggio N.. (2023). Supernatural explanations across 114 societies are more common for natural than social phenomena. Nat. Hum. Behav. 7, 707–717. doi: 10.1038/s41562-023-01558-0, PMID: 37012368

[ref68] Jawer M. A. Massullo B. Laythe B. Houran J. (2020). Environmental “gestalt influences” pertinent to the study of haunted houses. J. Soc. Psych. Res. 84, 66–92.

[ref69] Kandel N. (2020). Information disorder syndrome and its management. J. Nepal Med. Assoc. 58, 280–285. doi: 10.31729/jnma.4968, PMID: 32417871 PMC7580464

[ref70] Katz D. L. O’Connell M. Yeh M. C. Nawaz H. Njike V. Anderson L. M.. (2005). Public health strategies for preventing and controlling overweight and obesity in school and worksite settings: a report on recommendations of the Task Force on Community Preventive Services. MMWR Recomm. Reports. 54, 1–12., PMID: 16261131

[ref71] Kazak A. E. (2018). Journal article reporting standards [editorial]. Am. Psychol. 73, 1–2. doi: 10.1037/amp0000263 29345483

[ref72] Kincaid J. P. Fishburne R. P. Rogers R. L. Chissom B. S. (1975). Derivation of new readability formulas (automated readability index, fog count and Flesch Reading ease formula) for navy enlisted personnel. Millington, Tennessee: Naval Technical Training Command Research Branch.

[ref73] Ku K. Y. L. Fung T. M. Y. Au A. C. Y. Choy A. Y. O. Kajimoto M. Song Y. (2023). Helping young students cope with the threat of fake news: efficacy of news literacy training for junior-secondary school students in Hong Kong. Educ. Stud. 1, –19. doi: 10.1080/03055698.2023.2296345

[ref74] Kumar V. K. Pekala R. J. (2001). “Relation of hypnosis-related attitudes and behaviors to paranormal belief and experience: a technical review” in Lange Hauntings and poltergeists: Multidisciplinary perspectives. ed. Houran J. (Jefferson, North Carolina: McFarland & Co.), 260–279.

[ref75] Lam S. K. K. Cheung C. T. Y. Tong W. Chien W. T. Chiu C.-D. Van Emmerik A.. (2025). Effects of an online psychoeducational program for people with dissociative symptoms: a randomized controlled trial. Res. Soc. Work. Pract. doi: 10.1177/10497315251340902

[ref76] Lange R. Greyson B. Houran J. (2004). A Rasch scaling validation of a “core” near-death experience. Br. J. Psychol. 95, 161–177. doi: 10.1348/000712604773952403, PMID: 15142300

[ref77] Lange R. Houran J. (1999). The role of fear in delusions of the paranormal. J. Nerv. Ment. Dis. 187, 159–166. doi: 10.1097/00005053-199903000-00005, PMID: 10086472

[ref78] Lange R. Houran J. Dagnall N. Drinkwater K. Caputo G. B. (2023). Perceptual bandwagon effects with “deep” imaginary companions. J. Sci. Expl. 37, 602–615. doi: 10.31275/20232645

[ref79] Lange R. Thalbourne M. A. Houran J. Storm L. (2000). The revised Transliminality scale: reliability and validity data from a Rasch top-down purification procedure. Consciousn. Cogn. 9, 591–617. doi: 10.1006/ccog.2000.0472, PMID: 11150227

[ref9002] Langston W. Hubbard T. Fehrman C. D’Archangel M. Anderson K. (2020). The role of personality in having a ghost experience and the role of personality and experience in the development of ghost belief. Pers Individ Diff. 163:110077. doi: 10.1016/j.paid.2020.110077

[ref80] Laythe B. Houran J. Dagnall N. Drinkwater K. (2021). Conceptual and clinical implications of a “haunted people syndrome”. Spirit. Clin. Pract. 8, 195–214. doi: 10.1037/scp0000251

[ref81] Laythe B. Houran J. Dagnall N. Drinkwater K. O’Keeffe C. (2022). Ghosted ! Exploring the haunting reality of paranormal encounters. Jefferson, North Carolina: McFarland & Co.

[ref82] Laythe B. Houran J. Little C. (2021). The ghostly character of childhood imaginary companions: an empirical study of online accounts. J. Parapsychol. 85, 54–74. doi: 10.30891/jopar.2021.01.07

[ref83] Laythe B. Houran J. Ventola A. (2018). A split-sample psychometric study of haunters. J. Soc. Psych. Res. 82, 193–218.

[ref84] Letchford A. Moat H. S. Preis T. (2015). The advantage of short paper titles. R. Soc. Open Sci. 2:150266. doi: 10.1098/rsos.150266, PMID: 26361556 PMC4555861

[ref85] Lewandowsky S. Ecker U. K. H. Cook J. (2017). Beyond misinformation: understanding and coping with the “post-truth” era. J. Appl. Res. Mem. Cogn. 6, 353–369. doi: 10.1016/j.jarmac.2017.07.008

[ref86] Lincoln M. Lincoln B. (2015). Toward a critical hauntology: bare afterlife and the ghosts of Ba Chúc. Comp. Stud. Soc. Hist. 57, 191–220. doi: 10.1017/S0010417514000644

[ref87] Little C. Laythe B. Houran J. (2021). Quali-quantitative comparison of childhood imaginary companions and ghostly episodes. J. Soc. Psych. Res. 85, 1–30.

[ref88] Maher M. (2015). “Ghosts and poltergeists: an eternal enigma” in Parapsychology: A handbook for the 21st century. eds. Cardeña E. Palmer J. Marcussion-Clavertz D. (Jefferson, North Carolina: McFarland & Co.), 327–340.

[ref89] Maraldi E. O. (2017). Letter to the editor: the scientific investigation of anomalous self and identity experiences. J. Nerv. Ment. Dis. 205:900. doi: 10.1097/NMD.0000000000000762, PMID: 29077653

[ref90] McAndrew F. T. (2020). The psychology, geography, and architecture of horror: how places creep us out. Evol. Stud. Imag. Cult. 4, 47–62. doi: 10.26613/esic.4.2.189

[ref91] McClenon J. (2012). A community survey of psychological symptoms: evaluating evolutionary theories regarding shamanism and schizophrenia. Mental Health Relig. Cult. 15, 799–816. doi: 10.1080/13674676.2011.637913

[ref92] McCrudden M. T. Schraw G. (2007). Relevance and goal-focusing in text processing. Educ. Psychol. Rev. 19, 113–139. doi: 10.1007/s10648-006-9010-7

[ref93] McLaughlin G. H. (1969). SMOG grading: a new readability formula. J. Read. 12, 639–646.

[ref95] Mohr S. Huguelet P. (2004). The relationship between schizophrenia and religion and its implications for care. Swiss Med. Wkly. 134, 369–376. doi: 10.4414/smw.2004.10322, PMID: 15340880

[ref96] Moore D. W. (2005). Three in four Americans believe in paranormal: Little change from similar results in 2001. Washington, D.C.: Gallup News Service.

[ref97] Muris P. Merckelbach H. Ollendick T. H. King N. J. Bogie N. (2001). Children’s nighttime fears: parent–child ratings of frequency, content, origins, coping behaviors and severity. Behav. Res. Ther. 39, 13–28. doi: 10.1016/S0005-7967(99)00155-2, PMID: 11125721

[ref98] Nickell J. (2012). The science of ghosts: Searching for spirits of the dead. Amherst, New York: Prometheus.

[ref99] Nielsen J. Loranger H. (2006). Prioritizing web usability. Berkeley, California: New Riders.

[ref9004] O’Keeffe C. Houran J. Houran D. J. Drinkwater K. Dagnall N. Laythe B. (2019). The Dr. John Hall story: A case study of putative “haunted people syndrome.” Ment. Health Relig. Cult. 22, 910–929. doi: 10.1080/13674676.2019.1674795

[ref100] OpenAI. (2023). *ChatGPT-4 (GPT-4)* [computer software]. Available online at: https://www.openai.com/gpt-4 (Accessed January 03, 2025).

[ref101] Paiva C. E. Lima J. P. S. N. Paiva B. S. R. (2012). Articles with short titles describing the results are cited more often. Clinics 67, 509–513. doi: 10.6061/clinics/2012(05)17, PMID: 22666797 PMC3351256

[ref102] Palmer G. Hastings A. (2013). “Exploring the nature of exceptional human experiences: recognizing, understanding, and appreciating EHEs” in The Wiley-Blackwell handbook of transpersonal psychology. eds. Friedman H. L. Hartelius G. (Hoboken, New Jersey: Wiley Blackwell), 333–351.

[ref103] Palmer J. A. Honorton C. Utts J. (1989). Reply to the National Research Council study on parapsychology. J. Am. Soc. Psych. Res. 83, 31–49.

[ref104] Parsons S. T. (2015). Ghostology: The art of the ghost hunter. Surrey, United Kingdom: White Crow Books.

[ref105] Parsons S. T. (2018). Guidance notes for investigators of spontaneous cases: apparitions, hauntings, poltergeists and similar phenomena. London, United Kingdom: Society for Psychical Research.

[ref601] Peters E. Dieckmann N. Dixon A. Hibbard J. H. Mertz C. K. (2007). Less is more in presenting quality information to consumers. Med. Care Res Rev. 64, 169–190. doi: 10.1177/10775587070640020301, PMID: 17406019

[ref106] Pew Research Center. (2009). Many Americans mix multiple faiths. Available online at: https://www.pewforum.org (Accessed February 03, 2025).

[ref9005] Plante T. E. Schwartz G. E. (Eds.) (2021). Interaction with the divine, the sacred, and the deceased: Psychological, scientific, and theological perspectives. Routledge/Taylor \u0026amp; Francis. doi: 10.4324/9781003105749

[ref107] Playfair G. L. (1980). This house is haunted: The true story of the Enfield poltergeist. Surrey, United Kingdom: White Crow Books.

[ref108] Potts J. (2004). “Ghost hunting in the twenty-first century” in From shaman to scientist: Essays on humanity's search for spirits. ed. Houran J. (Lanham, Maryland: Scarecrow Press), 211–232.

[ref109] Python Software Foundation. (2023). *Python* (version 3.9.21) [programming language]. Available online at: https://www.python.org (Accessed February 03, 2025).

[ref110] Rabeyron T. (2022). When the truth is out there: counseling people who report anomalous experiences. Front. Psychol. 12:693707. doi: 10.3389/fpsyg.2021.693707, PMID: 35058829 PMC8764292

[ref9006] Rabeyron T. Loose T. (2015). Anomalous experiences, trauma, and symbolization processes at the frontiers between psychoanalysis and cognitive neurosciences. Front Psychol. 6:1926. doi: 10.3389/fpsyg.2015.01926 26732646 PMC4685320

[ref112] Reichheld F. F. (2003). The one number you need to grow. Harv. Bus. Rev. 81, 46–124. 14712543

[ref113] Roll W. G. (1977). “Poltergeists” in Handbook of parapsychology. ed. Wolman B. B. (New York: Van Nostrand Reinhold), 382–413.

[ref114] Rosen C. Park S. Baxter T. Tufano M. Giersch A. (2023). Sensed presence, attenuated psychosis, and transliminality: at the threshold of consciousness. Psychopathology 56, 359–370. doi: 10.1159/000528572, PMID: 36754040 PMC10534996

[ref115] Ross C. A. Joshi S. (1992). Paranormal experiences in the general population. J. Nerv. Ment. Dis. 180, 357–361. doi: 10.1097/00005053-199206000-00004 1593270

[ref116] Roxburgh E. C. Evenden R. E. (2016a). ‘They daren’t tell people’: therapists’ experiences of working with clients who report anomalous experiences. Europ. J. Psychother. Counsel. 18, 123–141. doi: 10.1080/13642537.2016.1170059

[ref117] Roxburgh E. C. Evenden R. E. (2016b). ‘Most people think you're a fruit loop’: clients’ experiences of seeking support for anomalous experiences. Counsel. Psychother. Res. 16, 211–221. doi: 10.1002/capr.12077

[ref118] Roxburgh E. C. Vernon D. Schofield M. B. (2024). Sensory processing sensitivity, transliminality, and boundary-thinness as predictors of anomalous experiences, beliefs, and abilities. Curr. Psychol. 43, 30098–30106. doi: 10.1007/s12144-024-06619-9

[ref119] Sanford J. R. (2016). Facing our demons: psychiatric perspectives on exorcism rituals. Hilltop Rev. 8, 87–93.

[ref120] Sangha L. (2020). The social, personal, and spiritual dynamics of ghost stories in early modern England. Hist. J. 63, 339–359. doi: 10.1017/S0018246X1800047X

[ref9003] Santos C. Michaels J. L. (2022). What are the core features and dimensions of “spirituality”? Applying a partial prototype analysis to understand how laypeople mentally represent spirituality as a concept. Psychol Relig Spiritual. 14, 10–20. doi: 10.1037/REL0000380

[ref121] Schulz K. F. Grimes D. A. (2005). Sample size calculations in randomised trials: mandatory and mystical. Lancet. 365, 1348–1353. doi: 10.1016/S0140-6736(05)61034-3, PMID: 15823387

[ref122] Scott B. (2024). About us. Available online at: https://readabilityformulas.com/about-us/ (Accessed January 03, 2025).

[ref124] Sharpley R. (2018). Tourism, tourists and society. 5th Edn. London: Routledge.

[ref125] Shojania K. G. Grimshaw J. M. (2005). Evidence-based quality improvement: the state of the science. Health Aff. 24, 138–150. doi: 10.1377/hlthaff.24.1.138, PMID: 15647225

[ref126] Siegel P. (1986). “Parapsychological counseling: six patterns of response to spontaneous psychic experiences” in Research in parapsychology 1985. eds. Weinger D. H. Radin D. I. (Lanham, Maryland: Scarecrow Press), 172–174.

[ref127] Simmonds-Moore C. A. (2024). Exploring the correlates and nature of subjective anomalous interactions with objects (psychometry): a mixed methods survey. Front. Psychol. 15:1365144. doi: 10.3389/fpsyg.2024.1365144, PMID: 39286561 PMC11404038

[ref123] Smith A. E. Senter R. J. (1967). Automated readability index. Springfield, Virginia: AMRL-TR. Aerospace Medical Research Laboratories. 5302480

[ref128] Storm L. Goretzki M. (2021). The psychology and parapsychology of spiritual emergency. J. Sci. Expl. 35, 36–64. doi: 10.31275/20211889

[ref129] Subotic S. Mukherjee B. (2014). Short and amusing: the relationship between title characteristics, downloads, and citations in psychology articles. J. Inf. Sci. 40, 115–124. doi: 10.1177/0165551513511393

[ref600] Sun Y. Zhang Y. Gwizdka J. Trace C. B. (2019). Consumer evaluation of the quality of online health information: systematic literature review of relevant criteria and indicators. J. Med. Intern. Res. 21:e12522. doi: 10.2196/12522, PMID: 31045507 PMC6521213

[ref130] Swami V. Pietschnig J. Stieger S. Voracek M. Tran U. S. (2024). Transliminality – converging evidence of associations with and openness to experience and its facets. Z. Psychol. 232, 269–278. doi: 10.1027/2151-2604/a000576

[ref131] Syroid N. D. Agutter J. Drews F. A. Westenskow D. R. Albert R. W. Bermudez J. C.. (2002). Development and evaluation of a graphical anesthesia drug display. Anesthesiology 96, 565–575. doi: 10.1097/00000542-200203000-00010, PMID: 11873029

[ref132] Tarlow P. (2005). “Dark tourism: the appealing ‘dark’ side of tourism and more” in Niche tourism: Contemporary issues. ed. Novelli M. (Burlington, Massachusetts: Trends and Cases), 47–57.

[ref133] Taves A. Barlev M. (2023). A feature-based approach to the comparative study of “nonordinary” experiences. Am. Psychol. 78, 50–61. doi: 10.1037/amp0000990, PMID: 35201784

[ref134] Thalbourne M. A. Crawley S. E. Houran J. (2003a). Temporal lobe lability in the highly transliminal mind. Personal. Individ. Differ. 35, 1965–1974. doi: 10.1016/S0191-8869(03)00044-8

[ref135] Thalbourne M. A. Houran J. (2005). Patterns of self-reported happiness and substance use in the context of transliminality. Personal. Individ. Differ. 38, 327–336. doi: 10.1016/j.paid.2004.04.011

[ref136] Thalbourne M. A. Houran J. Alias A. G. Brugger P. (2001). Transliminality, brain function, and synesthesia. J. Nerv. Ment. Dis. 189, 190–192. doi: 10.1097/00005053-200103000-00009, PMID: 11277357

[ref137] Thalbourne M. A. Houran J. Crawley S. E. (2003b). Childhood trauma as a possible antecedent of transliminality. Psychol. Rep. 93, 687–694. doi: 10.2466/pr0.2003.93.3.687, PMID: 14723429

[ref138] Tricco A. C. Langlois E. V. Straus S. E. (2017). Rapid reviews to strengthen health policy and systems: a practical guide. Geneva: World Health Organization.

[ref139] Van Dyke P. T. Juncosa M. L. (1973). Paranormal phenomena—briefing on a net assessment study. A working note prepared for the Advanced Research Projects Agency (WN-8019-ARPA). Available online at: https://www.esd.whs.mil/Portals/54/Documents/FOID/Reading%20Room/International_Security_Affairs/paranormal_briefing.pdf (Accessed January 03, 2025).

[ref140] Ventola A. Houran J. Laythe B. Storm L. Parra A. Dixon J.. (2019). A transliminal ‘dis-ease’ model of poltergeist ‘agents. J. Soc. Psych. Res. 83, 144–171.

[ref141] Wahbeh H. Delorme A. Radin D. (2023). Rating the persuasiveness of empirical evidence for the survival of consciousness after bodily death: a cross-sectional study. J. Anomal. Exp. Cogn. 3, 78–109. doi: 10.31156/jaex.24125

[ref142] Wardle C. Derakhshan H. (2017). Information disorder: toward an interdisciplinary framework for research and policy making Council Europe Available online at: https://edoc.coe.int/en/media/7495-information-disorder-toward-an-interdisciplinary-framework-for-research-and-policy-making.html (Accessed February 03, 2025).

[ref9007] Wilt J. A. Stauner N. May R. W. Fincham F. D. Pargament K. I. Exline J. J. (2022). Who engages with supernatural entities? An investigation of personality and cognitive style predictors. Imagin. Cogn. Pers. 41, 373–414. doi: 10.1177/02762366211065677

[ref144] Woods A. Wilkinson S. (2017). Appraising appraisals: role of belief in psychotic experiences. Lancet Psychiatry 4, 891–892. doi: 10.1016/S2215-0366(17)30434-0, PMID: 29179922

[ref605] YouGov. (2022). “Americans describe their paranormal encounters [Survey],” in *YouGov America*. Available online at: https://today.yougov.com/society/articles/44141-paranormalencounters-yougov-poll-october-12-2022

[ref145] Zingrone N. L. Alvarado C. S. Hövelmann G. H. (2015). “An overview of modern developments in parapsychology” in Parapsychology: A handbook for the 21st century. eds. Cardeña E. Palmer J. Marcusson-Clavertz D. (Jefferson, North Carolina: McFarland & Co), 13–29.

