# Correction: Quality-checking a novel “fact sheet” on ghostly episodes

**DOI:** 10.3389/fpsyg.2025.1678465

**Published:** 2025-08-28

**Authors:** Brandon Jon Massullo, James Houran, Alex Escolá Gascón, Ciarán O'Keeffe, Kenneth Graham Drinkwater, Neil Dagnall

**Affiliations:** ^1^Wooster Community Hospital, Wooster, OH, United States; ^2^Integrated Knowledge Systems, Dallas, TX, United States; ^3^Comillas Pontifical University, Madrid, Spain; ^4^Buckinghamshire New University, High Wycombe, United Kingdom; ^5^Manchester Metropolitan University, Manchester, United Kingdom

**Keywords:** encounter experiences, fact-checking, information sheet, public education, scientific literacy, sense-making

The reference for Carmen et al., 2013 was erroneously written as Carman, K. L., Dardess, P., Maurer, M., Sofaer, S., Adams, K., Bechtel, C., et al. (2014). Patient and family engagement: A framework for understanding the elements and developing interventions and policies. *Health Affairs, 33*, 223–231. doi: 10.1377/hlthaff.2013.1139

It should be Carman, K. L., Dardess, P., Maurer, M., Sofaer, S., Adams, K., Bechtel, C., et al. (2013). Patient and family engagement: a framework for understanding the elements and developing interventions and policies. *Health Affairs* 32, 223–231. doi: 10.1377/hlthaff.2012.1133

The reference for Castro et al., 2014 was erroneously written as Castro, M., Burrows, R., & Wooffitt, R. (2014). The paranormal is (still) normal: The sociological implications of a survey of paranormal experiences in Great Britain. *Sociological Research Online, 19*, 277–283. doi: 10.5153/sro.3441

It should be Castro, M., Burrows, R., and Wooffitt, R. (2014). The paranormal is (still) normal: the Sociological implications of a survey of paranormal experiences in Great Britain. *Sociol. Res. Online* 19, 30–44. doi: 10.5153/sro.3355

The reference for Katz et al. 2005 was erroneously written as Katz, D. L., Meller, S., and Williams, A. L. (2012). Public health strategies for preventing and controlling obesity in school and worksite settings. *Preventive Medicine, 55*, 260–S105. doi: 10.1016/j.ypmed.2012.06.012

It should be Katz, D. L., O'Connell, M., Yeh, M. C., Nawaz, H., Njike, V., Anderson, L. M., et al. (2005). Public health strategies for preventing and controlling overweight and obesity in school and worksite settings: a report on recommendations of the Task Force on Community Preventive Services. *MMWR Recomm. Reports* 54, 1–12.

The reference for Schulz & Grimes, 2005 was erroneously written as Schulz, K. F., & Grimes, D. A. (2005). Sample size calculations in randomised trials: Mandatory and mystical. *The Lancet, 365*, 1348–1353. doi: 10.1016/S0140-6736(02)07737-8

It should be for Schulz, K. F., and Grimes, D. A. (2005). Sample size calculations in randomised trials: Mandatory and mystical. *Lancet* 365, 1348–1353. doi: 10.1016/S0140-6736(05)61034-3

Miller and Reynolds, 2004 was erroneously cited in Introduction, Paragraph 3. This sentence previously stated:

“By summarizing key facts and presenting them in an organized way, information sheets simplify complex topics and enable users to better understand and remember pertinent data or associated recommendations (Miller and Reynolds, 2004)”

The corrected sentence appears below:

“By summarizing key facts and presenting them in an organized way, information sheets simplify complex topics and enable users to better understand and remember pertinent data or associated recommendations (Peters et al., 2007).

The citation and reference for Miller and Reynolds, 2004 has been removed.

The following citation and reference have been added:

Peters, E., Dieckmann, N., Dixon, A., Hibbard, J. H., and Mertz, C. K. (2007). Less is more in presenting quality information to consumers. *Med. Care Res Rev*. 64, 169–190. doi: 10.1177/10775587070640020301 was not cited in the article.

Redman et al., 2011 was erroneously cited in Introduction, Paragraph 3. This sentence previously stated:

And since Fact Sheets are often created by trusted experts or institutions, they are generally viewed as a reliable and valuable resource for education and advocacy (Redman et al., 2011).

The corrected sentence appears below:

And since Fact Sheets are often created by trusted experts or institutions, they are generally viewed as a reliable and valuable resource for education and advocacy (Sun et al., 2019).”

The citation and reference for Redman et al., 2011 have been removed.

The following citation and reference has been added:

Sun, Y., Zhang, Y., Gwizdka, J., and Trace, C. B. (2019). Consumer evaluation of the quality of online health information: systematic literature review of relevant criteria and indicators. *J. Med. Intern. Res*. 21:e12522. doi: 10.2196/12522

A correction has been made to Introduction, Paragraph 1. This sentence previously stated:

“Watt et al. (2015) noted that 12% of respondents had encountered unusual physical events they interpreted as poltergeist activity.”

The corrected sentence appears below:

“Some averaged statistics (Ross and Joshi, 1992; YouGov, 2022) suggest that approximately 12% of survey respondents had encountered unusual physical events they interpreted as poltergeist activity”

The citation and reference for Watt et al., 2015 has been removed.

The following citations and references have been added.

Ross, C. A., and Joshi, S. (1992). Paranormal experiences in the general population. *J. Nervous Ment. Dis*. 180, 357–368. doi: 10.1097/00005053-199206000-00004

YouGov. (2022). “Americans describe their paranormal encounters [Survey],” in *YouGov America*. Available online at: https://today.yougov.com/society/articles/44141-paranormal-encounters-yougov-poll-october-12-2022

The original version of this article has been updated.

