# Supplementary material for: Quality-checking a novel “fact sheet” on ghostly episodes
Source: Front Psychol. 2025 Jul 1;16:1585437. doi: 10.3389/fpsyg.2025.1585437 (PMC12259700; doi:10.3389/fpsyg.2025.1585437)
Supplement: Supplementary file 1 [file Supplementary_file_1.docx]

Appendix A. *Summary of Scientific Information on Ghostly Episodes for Information-Seekers and Information-Providers*

**Fact Sheet: “Ghostly Episodes at a Glance”**

***Are ghosts, hauntings, and poltergeists real?***

*Ghostly episodes* is a catch-all term for “ghosts, haunted houses, and poltergeist disturbances,” since growing evidence suggests that these events involve a common phenomenon or set of operating principles. Not all cases baffle modern science, but some episodes are sincerely reported and can deeply affect witnesses emotionally or psychologically. Many people’s accounts thus deserve to be taken seriously by scientists and studied further, if only to help afflicted people cope with these often-upsetting experiences. However, scientists hotly debate the nature of these anomalies. A few suggest that spirits might be involved, whereas most others think they represent the psychic abilities of living people who are unaware that they trigger the events. More skeptically-minded scientists speculate that natural causes are responsible, although the exact solution might not be proven or fully described at the time. Of course, scientists could discover that many factors are involved, or that there are sub- types of ghostly episodes with distinctly different sources. The general academic consensus, however, is that these experiences represent the actions or psychology of living people—not the work of “spirits or demonic forces.” But it remains a simple statement of fact that science currently lacks a comprehensive explanation for all aspects of sincerely reported ghostly episodes.

***Who experiences these phenomena?***

These episodes do not seem to occur randomly, but rather tend to be reported by individuals with natural or trained “hyper sensitivities” to their physical environments and own bodily functions. In fact, these people’s perceptions of external information and internal sensations can sometimes become blended or confused. It is also not surprising that these same people tend to have various other chemical, emotional, psychological, or social sensitivities. They also tend to report other types of mysterious experiences or events that seem unrelated to ghosts or poltergeist disturbances.

***Are these phenomena dangerous?***

It seems so, at least “sometimes” …although this answer has important caveats. Most often these episodes are psychologically distressing because they can be unpredictable or unmanageable. The mysterious nature of these episodes also causes some people to question their religious beliefs or their sense of reality and their place in it. However, though much rarer, episodes can also involve physical events like damage to certain objects or even scratches or cuts to witnesses’ bodies. Extreme cases have reportedly included objects being thrown around or odd fires starting. But more often than not, the immediate “danger” in ghostly episodes is minimal and confined to mental or spiritual anxiety.

***Can these phenomena be controlled or stopped?***

“Perhaps” is the most accurate answer, as particular interventions by paranormal investigators, religious leaders, or psychic mediums *occasionally* seem to work for certain people. One important analysis of 30 historical cases in which interventions were used found that success rates vary widely using methods like rituals, prayers, or blessings aimed at calming or dispelling the mysterious disturbances. Specifically, it was observed that 13% of time the phenomena ceased, 13% of the time there was temporary relief, 17% of the time the phenomena actually intensified, and 57% of the time there was no effect. But such tactics, even when successful, might be more about providing comfort and psychological support rather than directly addressing any supposed paranormal activity.

***What do skeptics say?***

Skeptics approach claims or beliefs with reasonable doubt and questioning rather than immediately accepting them as scientifically true. Such individuals with knowledge of these phenomena correctly caution that many cases have been traced to (a) fraud, (b) psychological factors like grief, expectation, or overactive imaginations, or (c) harmless misinterpretations or misunderstandings of mundane or unexpected naturally-occurring events. But many skeptics acknowledge that some cases can be difficult to explain with current scientific knowledge, although this could be due to insufficiencies with the methods used in investigations or the training of researchers assessing the information. Skeptics assess stories about the paranormal using the principle of “Occam’s Razor,” i.e., when you have two or more explanations for something, the simplest one is usually the best. Instead of complicating things with extra details, Occam’s Razor encourages us to choose the explanation that requires the fewest assumptions—it’s like saying, “Keep it simple!” So, if you hear mysterious “hoof beats” think horses, not unicorns.

***What should I do if my house seems haunted?***

This depends on what the goal is. Living with what seems to be a “ghost” is not always a problem; many people are simply intrigued by the idea or even think it’s fun. Such individuals often want simply to learn more about the phenomena, and below we explain how to satisfy this curiosity. Others might feel annoyed or perhaps threatened by the mysterious events. These people are encouraged to share their experiences with trusted, educated professionals like a local Psychology Professor or informed Clergy who usually will agree to discuss your concerns and possibly explore obvious explanations. For more intense cases, people can consult scientific organizations familiar with these phenomena about recommendations for on-site investigations, if appropriate. Two credible institutions are the *Society for Psychical Research* (https://www.spr.ac.uk/) in the U.K. and the *Parapsychological Association* (https://www.parapsych.org/) in the U.S. We generally encourage people not to request education or investigations from ghost-hunting groups or amateur paranormal researcher websites that have not been properly vetted by professional scientists in this field of study.

***Where can I find more reliable information?***

Many popular websites, books, magazines, TV documentaries, or online videos claim to provide scientific information about ghostly episodes. But these are generally unreliable sources of information because they often rely on anecdotal evidence, personal beliefs, or sensationalism rather than empirical evidence and academic rigor. Additionally, these resources usually prioritize entertainment value over factual accuracy, leading to exaggeration and embellishment of stories or research findings.

However, the good news is that many responsible scientists have studied and published research on ghostly episodes for over a century. Valuable summaries of this huge literature and the latest studies are readily available if you know where to look. The downside is that these other sources often use technical language or explanations. However, we recommended six academic resources to learn more about the science of ghostly episodes. One is a free online resource, whereas the others are books or chapters that either must be purchased via online bookstores or publisher websites, or borrowed from major public or university libraries:

- *Hauntings and Poltergeists: Multidisciplinary Perspectives*, edited by James Houran & Rense Lange (2001, McFarland & Co.)
- *Poltergeists*, by Alan Gauld & A. D. Cornell (2018, White Crow Books reprinting of 1979 edition)
- *Ghosted! Exploring the Haunting Reality of Paranormal Encounters*, by Brian Laythe, James Houran, Neil Dagnall, Ken Drinkwater, & Ciaran O’Keeffe (2024, McFarland & Co.)
- “Ghosts and Poltergeists: An Eternal Enigma” [book chapter, pp. 327–240] by Michaeleen Maher. In E. Cardeña, J. Palmer, & D. Marcussion-Clavertz (Editors), *Parapsychology: A Handbook for the 21st Century* (2015, McFarland & Co.)
- “Poltergeists” [book chapter, pp. 382–413] by William G. Roll. In B. B. Wolman (Editor), *Handbook of Parapsychology* (1977, Van Nostrand Reinhold).
- The Society for Psychical Research’s online “Psi Encyclopedia”: https://psi-encyclopedia.spr.ac.uk/. Searchable database filled with many educational entries. Search for keywords: “*apparition*, *ghost*, *ghost hunting*, *hauntings*, or *poltergeists*”

Appendix B. *Ghostly Episodes Fact Sheet Revised for Young Children (< 10 yrs old)*

**Ghostly Happenings: A Simple Guide for Kids**

**Are Ghosts Real?**

Some people believe in ghosts, while others don’t. Ghostly happenings include spooky things like haunted houses or objects moving by themselves. Scientists don’t agree on what causes these events. Some think it might be spirits, while others believe it’s just people’s minds playing tricks. We don’t have all the answers yet, but it’s an exciting mystery!

**Who Sees Ghosts?**

Ghostly happenings often happen to people who are extra-sensitive to their surroundings. These people might notice things that others don’t. They might also have strong feelings or other unusual experiences. It’s like having super-powered senses!

**Are Ghosts Dangerous?**

Most ghostly happenings aren’t dangerous, but they can feel scary. Sometimes, objects might move or even break, but this is rare. The biggest problem is that people might feel worried or confused. If you feel scared, talk to someone you trust.

**Can You Stop Ghostly Happenings?**

Sometimes people try to stop ghostly events with prayers, rituals, or blessings. These methods work for some but not everyone. It might help just to talk to someone who can listen and give advice.

**What Do Skeptics Think?**

Skeptics are people who ask lots of questions before believing something. They think many ghost stories are caused by tricks, imagination, or natural things like shadows or strange noises. They say it’s better to keep things simple—if you hear hoofbeats, think of horses, not unicorns!

**What Should I Do if I Think My House Is Haunted?**

If you think there’s a ghost in your house, don’t panic. Share your feelings with someone you trust, like a teacher or parent. Experts, like scientists or church leaders, might also help. Be careful about asking random ghost hunters, as not all of them are experts.

**Where Can I Learn More?**

Books and websites can teach you more about ghosts, but not all of them tell the truth. Look for trusted sources, like libraries or experts who study ghosts seriously. Some good resources include:

- **Hauntings and Poltergeists** by James Houran & Rense Lange
- **Ghosted! Exploring the Haunting Reality of Paranormal Encounters** by Brian Laythe and others
- The Society for Psychical Research’s online Psi Encyclopedia: https://psi-encyclopedia.spr.ac.uk

Remember, ghostly happenings are still a big mystery. It’s okay to be curious and ask questions. Who knows what we’ll learn in the future!
